# The Unsettled but Promising Interconnection of Human Rights, Mental Health, and Climate Change

**DOI:** 10.1017/jme.2025.10124

**Published:** 2025

**Authors:** Lance Gable

**Affiliations:** Wayne State University Law School, Detroit, United States

**Keywords:** human rights, mental health, climate change, global health, international law

## Abstract

Human rights courts may be on the cusp of recognizing linkages between the mental health impacts of climate change and human rights. However, several significant obstacles must be overcome before human rights protections are likely to be extended to cover the mental health impacts of climate change. Thus, the push for recognition of human rights protections for people facing mental health harms imposed by climate change must be pursued along with a multifaceted effort that employs regulatory and advocacy strategies alongside litigation, and more clearly establishes the interconnections between mental health, climate change, and human rights.

As the effects of human-induced climate change have continued to unfold, increased risks to mental health attributable to the current and future climate crisis have become a significant, although still considerably underappreciated, concern. Climate researchers have long predicted that more frequent environmental disruptions would increase levels of anxiety, depression, post-traumatic stress disorder (PTSD), suicidality, and other mental health conditions,^1^ and recent studies have supported this projection.^2^ The precarity and insecurity introduced by an uncertain climate future also contributes to mental health harms, with the Intergovernmental Panel on Climate Change concluding with “very high confidence” that “mental health challenges, including anxiety and stress, are expected to increase under further global warming in all assessed regions, particularly for children, adolescents, elderly, and those with underlying health conditions.”^3^

Despite this mounting evidence and attention, few governments have taken explicit action to address the mental health impacts of climate change.^4^ Where regulation and policymaking falter, litigation can attempt to fill the void and advance climate policies more protective of mental health. As Sam Varvastian outlines in his excellent article in this issue, “Climate Change and Mental Health: A Human Rights Perspective,” petitioners have increasingly invoked mental health in their claims before national and international human rights courts as justification for more aggressive state actions on climate change mitigation and adaptation.^5^ Yet these courts have hesitated to embrace the linkages between climate change, its mental health impacts, and human rights. Indeed, courts have been reticent to recognize that climate change policies implicate a right to health at all, much less a right to mental health. While Professor Varvastian hopefully suggests that human rights courts may be on the cusp of such a recognition, several significant obstacles must be overcome before human rights protections are likely to be extended to cover the mental health impacts of climate change. Consequently, the push for recognition of human rights protections for people facing mental health harms imposed by climate change must be pursued along with a multifaceted effort that employs regulatory and advocacy strategies alongside litigation, and more clearly establishes the interconnections between mental health, climate change, and human rights.

## Expanding research on the mental health effects of climate change

Researchers must continue to pursue studies that will augment the evidence base demonstrating the likely effects of climate change on health generally and mental health specifically.^6^ More rigorous and expansive research exploring connections between climate change and harms to mental health can better inform climate policymaking and may bolster public support for taking more concerted actions now to reduce these harms through climate mitigation and adaptation. Research that establishes a clearer link between climate change and mental health problems also may strengthen the causal arguments raised by petitioners in their human rights claims challenging inadequate climate policies. Yet scaling up research requires resources and prioritization from policymakers as well as the researchers themselves, which has presented an ongoing impediment despite growing attention. The most recent *Lancet* Countdown report on health and climate change notes that efforts to capture information about the mental health impacts of climate change “has so far been hindered by the persistent lack of standardised definitions, frequent stigmatisation and lack of recognition of mental health, and scarcity of globally relevant data on mental health impacts and care.”^7^ This relative dearth of investment in research will likely be perpetuated by current efforts to eviscerate federal funding for scientific research on climate change, public health, and mental health in the United States. It will be imperative for other institutions and funders to fill this gap and support this vital research.

## Recognizing the impact and importance of mental health

Policymakers worldwide must continue to highlight the importance of mental health as a core component of human health. Mental health has often received insufficient attention relative to physical health across numerous facets of law and policy, an oversight that often requires legislative or judicial interventions to remedy.^8^ This tendency to neglect consideration of mental health extends to human rights instruments, discourse, and litigation. Even human rights instruments and analyses that address the intersection of health and human rights primarily concentrate on physical health rather than mental health.^9^ Initiatives over the past decade to emphasize the importance of mental health in global health policy^10^ and to more clearly demonstrate the interconnection between mental health and human rights^11^ have had moderate success in bolstering the attention given to mental health concerns. But more progress must be made in breaking down the silos between physical and mental health in law and policy to ensure that mental health becomes a more significant factor in health policymaking, rather than an afterthought.

## Linking human rights, mental health, and climate change

The health implications of climate change have received increasing attention from human rights institutions, especially over the decade since the completion of the Paris Agreement in 2015. International human rights institutions have issued numerous resolutions, reports, and statements recognizing the link between human rights and climate change, including the United Nations Human Rights Council, the United Nations General Assembly, and Special Rapporteurs on the environment, health, food, Indigenous peoples, migrants, and water and sanitation, as well as the recently-created Special Rapporteur on Climate Change.^12^ Climate litigants, likewise, have increasingly incorporated rights-based arguments into their cases.^13^ However, as Professor Varvastian notes, these developments have not resulted in clear or widespread recognition by human rights courts that the mental health impacts of climate change violate human rights protections.^14^

Rights-based approaches have great promise for advancing policies protective of mental health.^15^ Human rights systems delineate obligations on states to protect, respect, and fulfill rights, including the right to health. States are also the only actors that have the capacity to advance meaningful efforts to combat the climate crisis. Thus, human rights arguments may provide a particularly effective means of compelling greater state action on climate change. And health-related arguments may provide a particularly compelling route to demonstrating the human rights harms of climate change.^16^

Nevertheless, rights-based approaches and the systems that enforce them have significant limitations. Human rights litigants face the typical litigation challenges of jurisdiction, standing, and causation. Indeed, several of the unsuccessful human rights claims described by Professor Varvastian failed when courts determined that the petitioners lacked standing or had brought untimely cases to international tribunals before fully exhausting domestic remedies.^17^ These procedural hurdles are compounded by the reluctance of many human rights courts to broadly recognize a lack of substantive health protections, including the potential for future harm to health, as human right violations.^18^ In this context, research demonstrating the scope of current mental health impacts — among them anxiety, stress, and depression — arising from concern about the future effects of climate change may provide persuasive evidence of causation to support claims of human rights violations.

## A multifaceted effort

Rights-based litigation pursued at the national, regional, and international levels may yet provide precedential and persuasive rulings holding the mental health effects of climate change to be human rights violations.^19^ Pending advisory opinions from the Inter-American Court of Human Rights^20^ and the International Court of Justice^21^ may provide the occaision to more directly connect human rights and climate-related harms. Litigation can be impactful, but is only one of several concurrent strategies necessary to mitigate the mental health harms of the climate crisis.^22^ Human rights institutions and global health actors have the opportunity to further elucidate the linkages between human rights, mental health, and climate change through the further strengthening of the legal determinants of health at all levels.^23^ Regulatory and legislative initiatives at national and subnational levels and a multiplicity of advocacy efforts must accompany litigation, institutional reports, and supportive research. In sum, human rights litigation to protect mental health and combat the climate crisis must be but one component of a broader, multipronged effort that transcends specific jurisdictions, institutions, and tactics. If successful, these interconnected efforts may spur more proactive laws and policies to avert the worst mental health effects of climate change and put us on a path to a more sustainable and livable future.
